# Effects of the Qinling‐Daba Mountains as Ecological Corridor on Patterns of Plant Distribution

**DOI:** 10.1002/ece3.71633

**Published:** 2025-07-07

**Authors:** Ya Jiang, Baiping Zhang, Yonghui Yao, Jiayu Li, Junjie Liu

**Affiliations:** ^1^ State Key Laboratory of Resources and Environmental Information System Institute of Geographic Sciences and Natural Resources Research Beijing China; ^2^ University of Chinese Academy of Sciences Beijing China

## Abstract

The Qinling‐Daba Mountains (QDM), extending east–west in central China, span warm temperate and subtropical zones and are characterized by complex geographical transitions and high biodiversity. They actually also act as a significant ecological corridor between the Tibetan Plateau and East China plains, but this almost has not been addressed. This study uses plant species data of 40 national nature reserves within QDM and 18 in adjacent area, performs consensus clustering at the levels of species, genus, and areal type, traces the origins and dispersal routes of 89 Chinese endemic genera, and, finally, assesses the importance and areal differentiation of environmental factors on species distribution. The results show: (1) The QDM as a corridor contribute greatly to the high biodiversity in the study areas, particularly in the easternmost and westernmost sections. (2) The QDM promote species interactions and exchanges between west China and east China. The genera involved are classified into four main types of geographic origins: Central‐East China components (41 genera) and North China components (8 genera) spread southwestward; Southwest components (24 genera) spread eastward and northeastward; while Northwest components (6 genera) show limited eastward spread. (3) Multi‐year average precipitation, elevation, and coldest quarter temperature significantly influence plant distribution. (4) Elevation differences (peak, base, and average) under 1000 m among reserves enhance plant dispersal, resulting in obvious corridor effect. This study provides theoretical support for understanding the corridor effect in the study area and its contribution to biodiversity pattern of China.

## Graphical abstract

The Qinling‐Daba Mountains serve as a complex transition zone with high biodiversity. They actually also act as a significant ecological corridor between the Tibetan Plateau and the East China plains, but this almost has not been addressed. This study aims to investigate the corridor effect in the study area and its contribution to the biodiversity pattern of China.

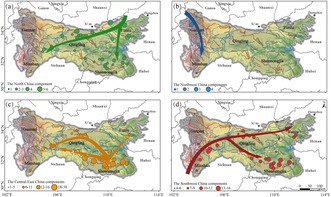

## 1 Introduction

The Qinling‐Daba Mountains (QDM) are composed of two parallel east–west extending mountain ranges located in central China, spanning the warm temperate and subtropical zones and with obvious geographical transitions, environmental complexity, high‐degree biodiversity, and climate sensitivity (Zhang 2019). Traditionally, the QDM are regarded the dividing line of northern and southern China, and recently the north–south transitional zone of China. Previous studies have been focused on their north–south differentiation, for example, climate factors (Fang 2001; Li et al. 2021; Zhu 1958), vegetation types (Wang et al. 2010; Zhang et al. 2021; Zhang and Zhang 1979), vertical vegetation zones (Fang and Gao 1963; Li et al. 2023; Zhao et al. 2019), and phenological shifts (Hu et al. 2023). However, this area is not a mere line; rather, it's a mountainous region characterized by high heterogeneity and multidimensional zoning (Zhang 2019). Mountains are considered as biodiversity hotspots, refuges, and diversification centers of endemism (Myers et al. 2000; Ying 2001). Actually, the QDM are also the only mountain ranges connecting the western plateaus and eastern plains in central China; however, this structure of “bridge” and its significance have seldom been addressed before.

Scholars have proposed diverse perspectives on the influence of mountains on species distribution (Badgley 2010; Cai et al. 2018; Heaney 2000; Steinbauer et al. 2016). Bănărescu suggested that mountains often act as barriers to biological dispersal (Banarescu 1992), with montane biotic communities exhibiting discontinuous distribution patterns, separated into distinct subunits (Whittaker et al. 2008). For example, the genetic structure of species such as *Cicerbita alpina* and *Ranunculus platanifolius* revealed long‐term isolation between eastern and western populations in the Carpathians (Stachurska‐Swakoń et al. 2011, 2020). The Southern Alps (in New Zealand), as a barrier to species migration, contributed to the allopatric speciation of *Galaxias paucispondylus* and *G. divergens* . Mayr pointed out that while the rise of major mountain ranges may hinder biological dispersal, it can simultaneously promote the formation of new species (Mayr 1963). Other scholars (Chester et al. 2013; Rahbek et al. 2019b) proposed that one of the impacts of mountains on biodiversity is their role as corridors, facilitating species migration and providing habitats. For instance, the Andes Mountains allow for the spread of tropical plants like the Rubiaceae family from the northern Andes to the south (Antonelli et al. 2009; Luebert and Weigend 2014). Similarly, the Rocky Mountains act as a north–south corridor, offering migratory routes for species during the Late Glacial period (Heintzman et al. 2016). The complex topography and relatively stable climatic conditions of the east–west oriented Alps provided a refuge and dispersal path for trees during the Fourth Glacial Period, thus promoting species differentiation in mountainous areas (Bennett et al. 1991; Jardim de Queiroz et al. 2022). The diversity of these perspectives stems from the complexity of mountain ecosystems. Large mountain ranges often feature diverse habitats, and their distinct orientations influence different species and communities (Chester 2012). The role of mountains in species and population distribution is dual in nature—they can act as ecological corridors facilitating biological dispersal or as barriers hindering it. This intricate mechanism makes mountains uniquely valuable for biogeographical research.

In recent years, some researches have begun to explore the corridor effects and connectivity of the QDM (Yu et al. 2021). Some studies have ever identified the Qinling Mountains as a crucial corridor for plant dispersal for a few special species in China, with four dispersal routes (Matuszak et al. 2016; Wang 1989a, 1992; Wang and Zhang 1994a, 1994b): (1) From the Hengduan Mountains in southwestern China through the Qinling Mountains toward the east or northeast of China, including such species as *Corallodiscus lanuginosu* and *Anemone tomentosa*. (2) From Yunnan‐Guizhou‐Sichuan region through the Qinling to the east or northeast of China, for species such as *Cunninghamia lanceolata* and *Tetrapanax papyrifer* . (3) From the Himalayas eastward through the Qinling Mountains to Northeast and East China include species such as *Lysionotus*, *Clematis puberula* var. *ganpiniana*, and *Caulophyllum robustum*. Some species have a disjointed distribution between the Himalayas and the Qinling, including *Hemiphragma heterophyllum* and *Piptanthus*. (4) From East China and Central China westward via the Qinling to North China are a few rare and protected species like *Fortunearia sinensis* and *Hemiptelea davidii*. These studies involved only plant origins and differentiation from a botanical perspective, and provided very limited understanding of the vegetation distribution patterns and the effect of the QDM as an ecological corridor.

This study utilized plant species data from 58 national nature reserves to explore the distribution patterns of plant species diversity in the QDM and adjacent regions; by tracing the origins and dispersal routes of Chinese endemic genera, to explore the role of the QDM as a migration corridor for species; and through random forest modeling and variance analysis, to evaluate the influence of environmental factors, particularly mountain topography, on vegetation migration and distribution. These efforts aim to deepen the understanding of plant distribution patterns in the QDM and their effect as an ecological corridor on biodiversity.

## 2 Data and Method

**FIGURE 1:**
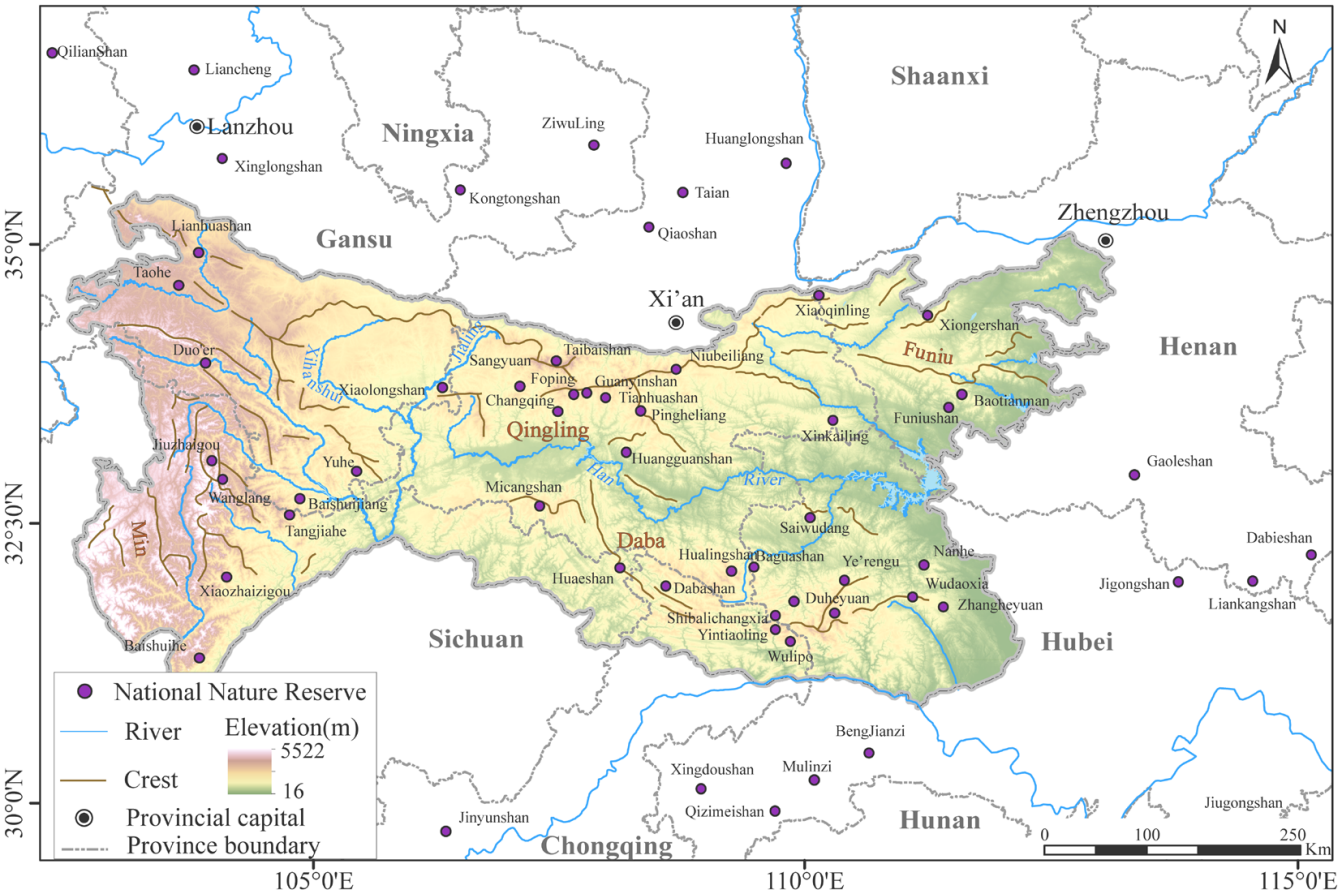
Locations of nature reserves in the Qinling‐Daba Mts.

The plant species database, based on determined scientific investigation reports for 40 national nature reserves in the Qinling‐Daba Mountains (QDM) and 18 adjacent reserves, spans 102° E–116° E and 29° N–37° N (Figure 1) (Zhang et al. 2022), with a total of 372 introduced cultivated species that were excluded (https://www.iplant.cn/). This database for the QDM and adjacent areas includes a total of 9491 seed plant species, belonging to 1729 genera and 211 families, with 3590 Chinese endemic species. Within the QDM, 8445 species of seed plants are recorded, belonging to 1602 genera and 202 families, with 3267 Chinese endemic species.

### 2.1 Geographic Types of Plant and Origin of the Chinese Endemic Genera

The remaining seed plants were phytogeographically classified into 15 geographic types based on Prof. Zhengyi Wu's plant classification system (including Chinese endemic genera and other types; see Table S1 for more details) (Wu 1991). Then, based on previous phylogenetic evidence and historical biogeography literature (Hao 1997; Li 1996; Wang 1979; Wang 1989a; Wang and Zhang 1994a, 1994b; Wang 1989b; Wu et al. 2005; Zhang 1995, 1996; Zhou and Arata 2005), the origin and diversification centers of the 89 Chinese endemic genera found in the nature reserves of the QDM were systematically identified one by one. When analyzing the distribution patterns of Chinese endemic genera in nature reserves, it is essential to consider different species within the same genus separately. For instance, the genus *Biondia* includes *Biondia chinensis* originating from Qinling, and other species from Sichuan, like *Biondia henryi*, *Biondia Pilosa*. The genus *Deinocheilos* includes species endemic to Jiangxi and the Three Gorges area (See Table S1 for classification details and reference). These genera can be categorized into six geographical components according to their origins and centers of evolution (Table 1, details see Table S1), while six genera remain of uncertain origin and require further investigation.

**TABLE 1:** Geographical components of Chinese endemic genera in the Qinling‐Daba Mts. national nature reserves.

Geographical components	Number of endemic genera	Proportion
Central‐East China component	41	46.07%
Southwest China component	24	26.97%
North China component	8	8.99%
Northwest China component	6	6.74%
Qinling component	3	3.37%
South China component	1	1.12%
Uncertain^a^	6	6.74%
Total	89	100%

^a^ “Uncertain,” primarily concentrated in southern China, and it has not been confirmed to have a distinct origin.

### 2.2 Environmental Variables

We selected environmental variables representing critical ecological gradients that influence plant distribution and richness (Antonelli et al. 2018; Godinho and Da Silva 2018; Hewitt 2004; Theodoridis et al. 2017). These variables include annual precipitation, annual mean temperature, mean temperature of coldest quarter, mean temperature of warmest quarter from the Last Glacial Maximum climate data and current climate data (2001–2017) interpolated using the ANUSPLIN method based on records from 89 climate stations in the study area (Liu et al. 2023). Additionally, elevation for each reserve, including the lowest point (base elevation), the average elevation, and the highest peak (peak elevation), were derived from the SRTM 90‐m resolution digital elevation model.

### 2.3 Classification of Nature Reserves

Nature reserves were classified into different regions using consensus clustering, based on genus‐level similarity (Szymkiewicz coefficient), species‐level similarity (Sørenson coefficient) and areal‐type similarity (Jaccard coefficient) (Shen et al. 2017). The similarity coefficients were converted into three‐level distance matrices for the consensus clustering process (López et al. 2008). This classification served as the foundation for delineating regions within the nature reserves, aiming to identify variations in plant distribution patterns and environmental conditions among these regions.

### 2.4 Mapping Chinese Endemic Genera

After categorizing the 89 Chinese endemic genera into different geographical components, we quantified the number of these genera of each component present in various nature reserves. This quantitative data was visualized using ArcGIS 10.7, resulting in a point‐based distribution map. The size of each point on the map corresponds to the abundance of genera within each component across the reserves. We then utilized the origin center of Chinese endemic genera as starting points to map potential species dispersal routes based on their distribution across different nature reserves.

### 2.5 Environmental Factor Analysis

We employed random forest method to calculate the mean decrease in accuracy of environmental factors, providing a measure of their relative importance in shaping species distribution patterns in the QDM (Terrer et al. 2021). Additionally, variance analysis was employed to test for significant differences in environmental factors among reserves across different regions. Depending on the homogeneity of variance, either ANOVA or the Kruskal–Wallis test was used to compare significant differences in environmental variables among regions.

Specifically, elevation differences among nature reserves within each region were further analyzed. Plots were used to visualize the range of base elevation, peak elevation, and mean elevation of nature reserves within the same region, as well as to compare these elevation variables between different regions. Based on the clustering results of the nature reserves, we statistically analyzed the elevation differences of nature reserves within and between regions to explore how mountain range influences plant distribution. All analyses were performed using R (v.4.4.0).

## 3 Results

### 3.1 Species Composition Plant Distribute Pattern and Classification of Nature Reserves

In the Qinling‐Daba Mountains (QDM), the 10 most species‐rich families are Rosaceae, Salicaceae, Ericaceae, Lauraceae, Fabaceae, Fagaceae, Berberidaceae, Sapindaceae, Oleaceae, and Rhamnaceae. The 10 most species‐rich genera are *Salix*, *Rubus*, *Berberis*, *Rosa*, *Acer*, *Spiraea*, *Euonymus*, *Ribes*, and *Viburnum*. The seed plant genera in the QDM belong to 15 areal types. The most common is the North Temperate element, accounting for 25.34% of the total genera. Its proportion exceeds 40% in the northwestern reserves but decreases to less than 20% toward the southeast. Next is the East Asian element, accounting for 12.68% of the total, with the Sino‐Himalayan subtype and the Sino‐Japanese subtype accounting for 3.07% and 4.02%, respectively. The former is mainly distributed to the west of Hanzhong, while the latter is to the east. Additionally, the Tropical element accounts for 12.39% of the total genera, constituting over 10% in the southeastern region of the Qinling Mountains but less than 5% in the northwestern alpine regions, such as Gansu and western Sichuan.

**FIGURE 2:**
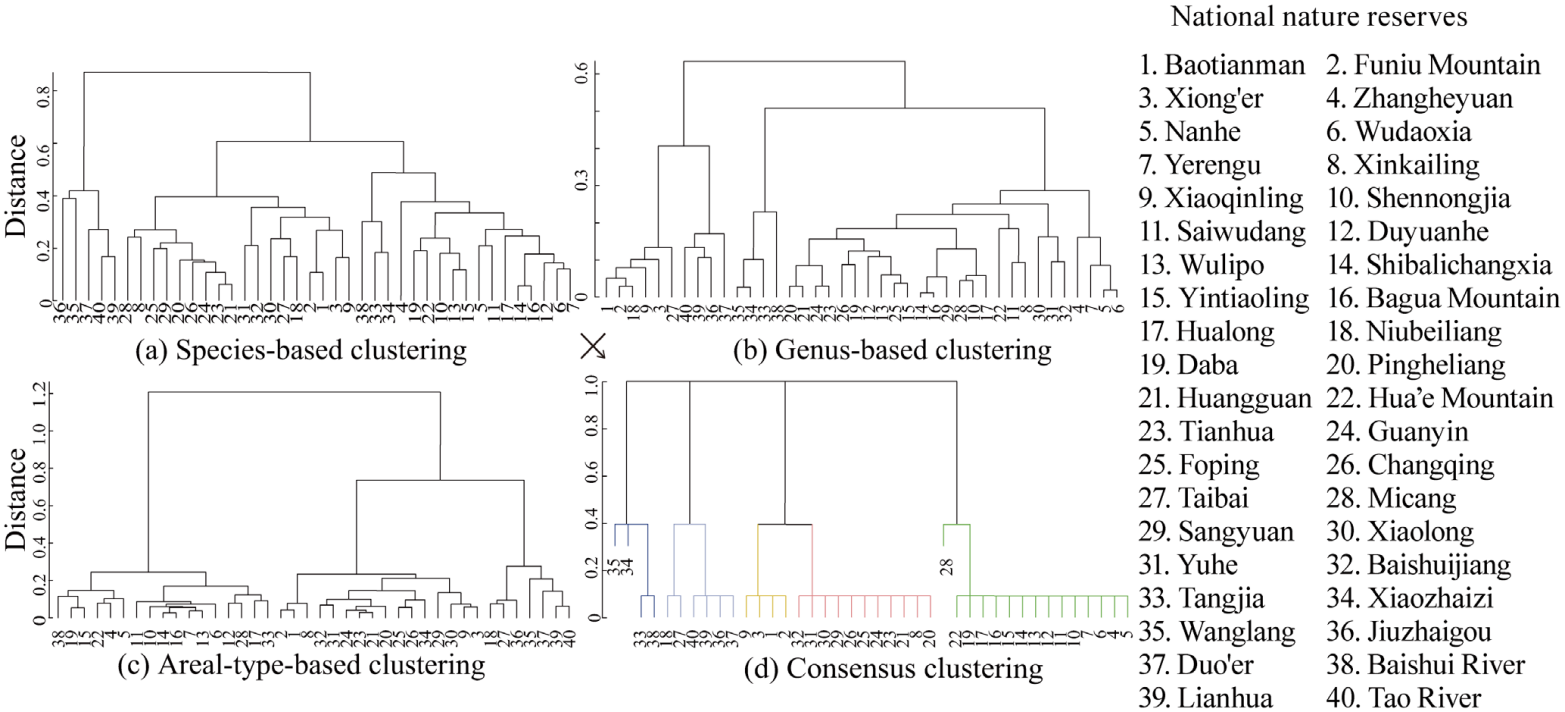
Cluster analysis of the Qinling‐Daba Mts. nature reserves based on (a) species, (b) genera, (c) areal type, and (d) consensus clustering results.

**FIGURE 3:**
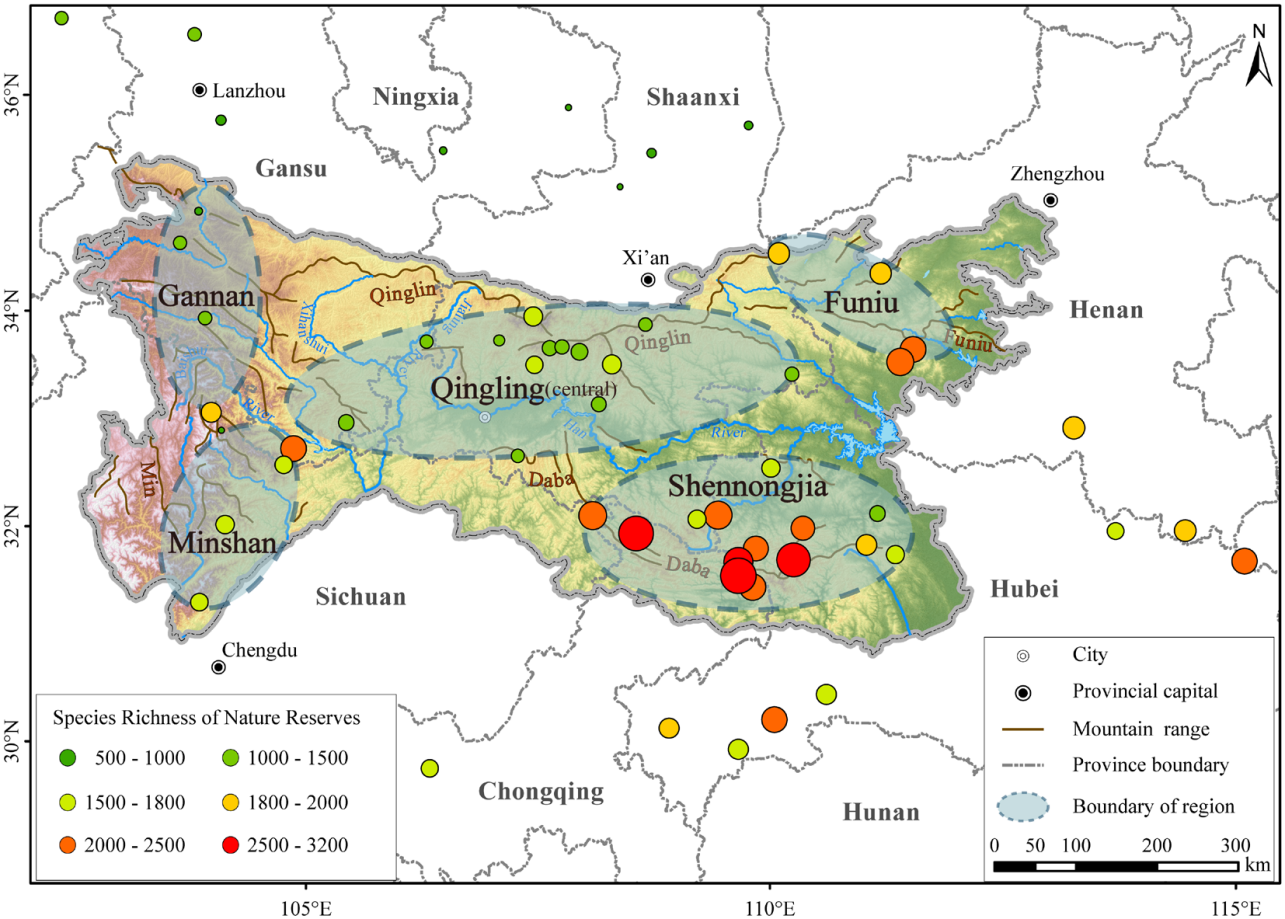
Species richness of nature reserves and their groups in the Qinling‐Daba Mts/adjacent areas.

The 40 reserves in the QDM were classified based on areal types, genus and species of seed plants, yielding the following results (Figure 2): (1) Species‐based clustering shows an east–west differentiation with the Jialing River as the border, and the eastern part can be identified by north–south differentiation with the Han River as the dividing line. (2) Genus‐based clustering reveals a noticeable difference between the northern and southern slopes of the Qinling Mountains, with further east–west differentiation along the Jialing River in the southern region. (3) Areal‐type‐based clustering shows a northwest–southeast difference demarcated by the Min‐Jialing‐Han River. These three clustering results demonstrate a high degree of consistency across the different levels of classification, indicating common spatial patterns in the floristic composition of the reserves. Using a consensus matrix, reserves classified under different clustering criteria were integrated into unified categories. The consensus clustering analysis ultimately divided the 40 reserves into five distinct regions (Figure 3): the Shennongjia region, the Funiu Mountains region, the Qinling (central) region, the Minshan region, and the Gannan (southern Gansu Province) region.

The species richness within nature reserves of the QDM surpasses that of adjacent areas. Most nature reserves in the QDM host over 2500 species of higher plants, whereas reserves in the northern vicinity generally contain only 500 to 1000 species. Within the QDM, the distribution pattern of plant biodiversity shows a trend of higher diversity at the eastern and western ends (Shennongjia, Funiu Mts. and Minshan regions) compared to the central areas. Taibai Mountain, the highest peak in the central Qinling region, has a significant elevation difference of 2600 m and distinct climatic variations between its northern and southern flanks. However, we found that Taibai Mountain nature reserve supports less biodiversity than lower‐altitude reserves at the same latitude in the eastern and western regions of QBM, such as Baotianman and Funiu Mountain. This pattern is consistent across different taxonomic levels (family, genus, and species) and for Chinese endemic taxa (Table 2).

**TABLE 2:** Comparison of species richness in the Eastern, Central, and Western nature reserves of the Qinling‐Daba Mts.

Region	Western		Central	Eastern	
Nature reserve name	Baishuijiang	Tangjiahe	Taibai	Baotianman	Funiu
Families	152	142	130	155	156
Genera	781	669	605	847	846
Species	2321	1557	1742	2263	2455
Chinese endemic species	842	522	636	591	707
Chinese endemic genera	40	24	24	26	34

### 3.2 Geographical Components and Dispersal Routes of Chinese Endemic Genera

**FIGURE 4:**
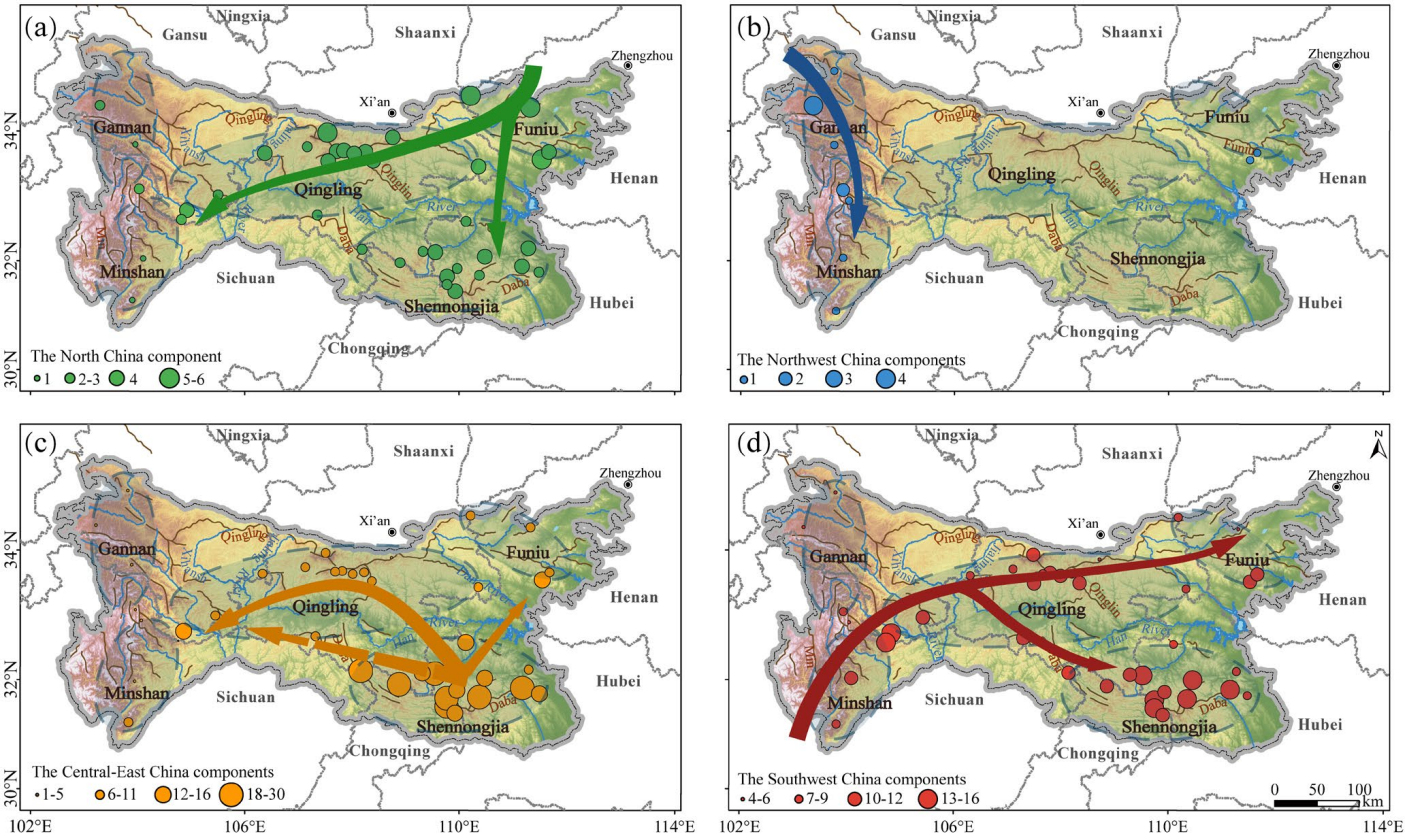
Distribution patterns of geographical components of Chinese endemic genera in the Qinling‐Daba Mts. (a) The North China components. (b) The Northwest China components. (c) The Central‐East China components. (d) The Southwest China components.

The most prominent four geographical components are Central‐East China, Southwest China, North China, and Northwest China The analysis of the distribution of Chinese endemic genera reveals distinct directional and radiative dispersal trends (Figure 4).

1. The predominant component among these endemic genera is the Central‐East China component, which includes 41 genera (46.07%) such as *Metasequoia*, *Triaenophora*, and *Deinocheilos*. The Central‐East China component spreads southwestward, with its proportion significantly decreasing. In the Daba‐Shennongjia region, this component can account for over 50% of all Chinese endemic genera, particularly in the Shennongjia reserve, where up to 30 genera are present. However, in other nature reserves located in the Gannan region, only 1–2 genera appear from this component.
2. Next is the Southwest China component, consisting of 24 genera (26.97%) originating from the Hengduan Mountains or Yunnan‐Guizhou‐Sichuan area, such as *Clematoclethra*, *Anemoclema*, and *Nannoglottis*. The Southwest component enters the QDM from the Minshan Mountains, constituting 45%–55% of the Chinese endemic genera in the western reserves. Its proportion decreases to 30%–40% as it spreads eastward. In terms of genus proportion and species abundance, this component shows strong spreading capacity along this route, with a weak attenuation trend.
3. The North China component includes eight genera (8.99%), such as *Opisthopappus*, *Myripnois*, and *Pteroxygonum*. The Northwest component, primarily originating from the Gansu‐Qinghai‐Tibet region, includes six genera (6.74%), such as *Metaeritrichium*, *Przewalskia*, and *Xanthopappus*. The North China component originates from regions such as Inner Mongolia, Liaodong, and the Taihang Mountains. Eight genera have entered the QDM via the Funiu Mountains, spreading primarily to the southwest with diminishing presence, and are entirely absent in northwestern areas such as Wanglang and Yuhe reserves.
4. The Northwest component is concentrated in the western Qinling region, particularly in the Minshan Mountains and Gannan areas. However, their eastward dispersal is highly limited. For instance, only one species, *Kengyilia hirsuta*, native to the northwest, unexpectedly appears in Funiu Mountains and Baotianman reserves, indicating rare eastward expansion.

Additionally, only three genera (3.37%) originate from the Qinling region, including *Biondia*, *Kungia*, and *Archakebia*, while one genus, *Tutcheria*, belongs to the South China component. These components are scarcely represented in the QBM, lacking distribution patterns.

### 3.3 The Influence of Environmental Factors on Species Distribution

**FIGURE 5:**
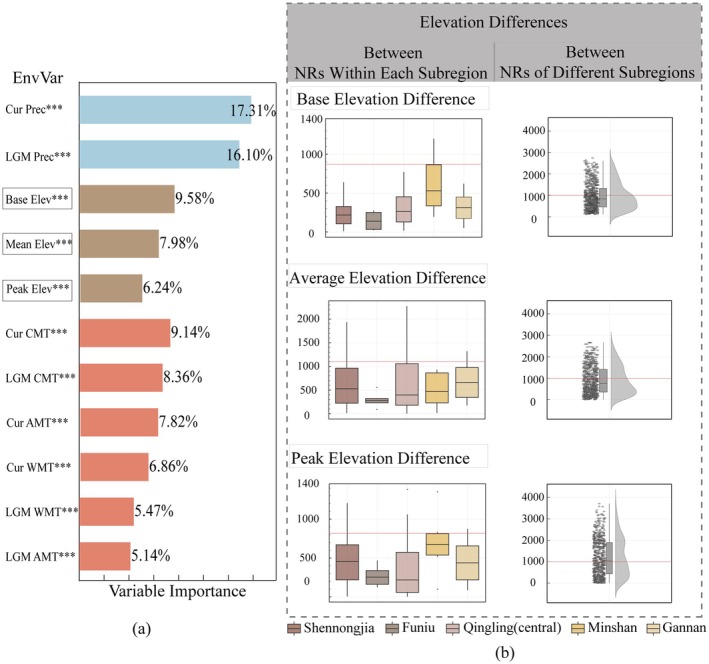
(a) Relative importance of environmental factors on plant distribution in the Qinling‐Daba Mts. (*** indicates significant differences between regions, *p* < 0.01). (b) Comparative statistical analysis of elevation differences of nature reserves within and between regions, including base, peak, and average elevations.

Environmental factors are the driving forces behind vegetation differentiation. This study reveals that precipitation, terrain and temperature show quite different for the five regions identified above (Figure 5a). Among these factors, annual precipitation plays a dominant role in the grouping of reserves, with current precipitation averages and Last Glacial Maximum (LGM), contributing 17.31% and 16.10% relative importance to plant distribution, respectively. Elevation differences among regions were statistically analyzed by considering the highest peak, the lowest point (base elevation) and the average elevation of each reserve. The base elevation and the average elevation contribute 9.58% and 7.98%, respectively, to species distribution, only next to precipitation. Among temperature‐related factors, the mean temperature of the coldest quarter during the current period (9.14%) and during the LGM (8.36%) is relatively influential, suggesting that extreme cold temperatures may be key constraints on species distribution.

Elevation differences among nature reserves were further analyzed. The results show that (Figure 5b): (1) The difference in base elevation between any two reserves within the same region is less than 900 m. (2) The difference in average elevation between reserves within the same region is generally less than 800 m, although a few reserves in the Qinling region exhibit differences of up to 1200 m. (3) The difference in peak elevation between reserves within the same region is less than 1000 m.

## 4 Discussion

### 4.1 Dispersal Routes of China Endemic Genera

**FIGURE 6:**
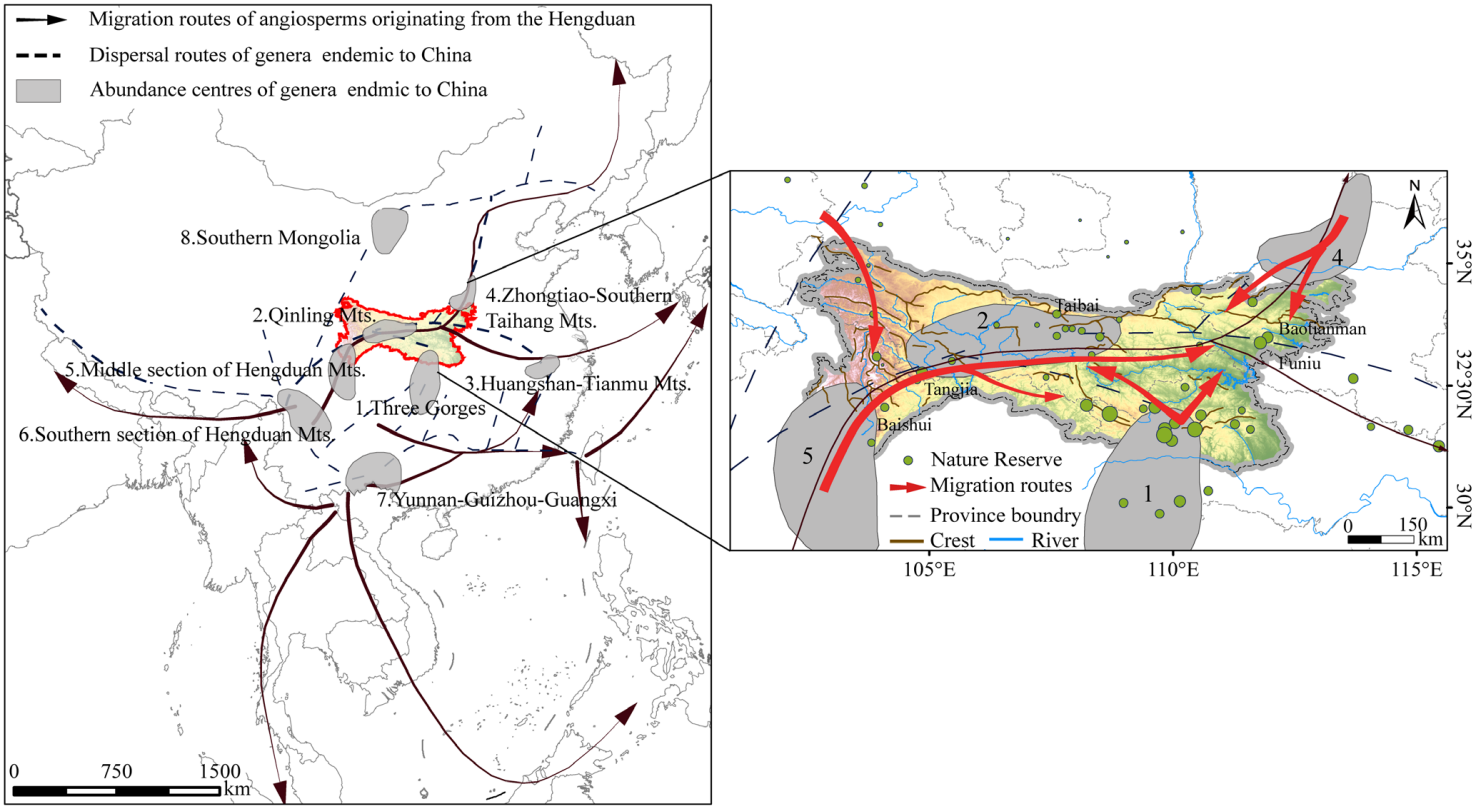
(a) Abundance centers and dispersal routes of China Genera endemic (Wang and Zhang 1994b) and the migration routes initiated and extending from Hengduan Center (Wang 1992). (b) Potential migration routes of species in the Qinling‐Daba Mts.

Based on the origins and distribution patterns of 89 Chinese endemic genera within the Qinling‐Daba Mountains, we proposed four potential species dispersal routes. Our basic species data were primarily derived from a seed plant database collected by Zhang et al. (2022) for almost all nature reserves in the study region, and the findings notably align with the migration routes suggested geographically and/or phylogenetically by Wang (1992) and Wang and Zhang (1994b) as shown in Figure 6.

Other studies, using data from a limited number of reserves by Shi (1998), Ding and Lu (2006), and Shi et al. (2021) and characterizing QDM plant geographical components by “north–south transition and east–west compatibility,” could also confirm our findings. Central China has been considered a refuge and preservation area for many ancient gymnosperm groups from the Neogene to Quaternary glaciations (Wu et al. 2005). The Central‐East China components, as the most species‐rich one, include 41 genera spreading southwestwards along the Qinling Mountains and appear in every reserve. The North China components, entering the QDM via the Funiu Mountain, are found in all regions except the northwest alpine areas. The Northwest China components, originating from the Gansu‐Qinghai‐Tibet region, spread mostly to Gannan and Minshan Mts. The Southwest China components, originating from the Hengduan Mountains and Yunnan‐Guizhou‐Sichuan region, primarily spread eastwards and northeastward with an obvious and strong trend. Unlike the radiating decline in abundance observed for other components, this geographic pattern of species dispersal revealed is likely due to the relatively young and highly differentiated flora of the Hengduan Mountains (Ying and Zhang 1984). However, the specific reasons remain to be further investigated.

### 4.2 Corridor or Barrier Effects of Mountains for Plants Spreading?

Mountain regions harbor roughly 87% of terrestrial global biodiversity, but solving the conundrum of the extraordinary diversity of mountains remains one of the greatest challenges (Rahbek et al. 2019a). Our findings suggest that the corridor effect of mountains could partially explain the biodiversity patterns observed in the QDM.

#### 4.2.1 Corridor Effect on Biodiversity

Our results indicate that the QDM hosts a much greater biodiversity than the neighboring areas. We propose that this high biodiversity is largely due to the QDM serving as a corridor for species dispersal. The Shennongjia region, located in the southeastern QDM, is considered a cradle of the World's Temperate flora (Wulff 1944; Ying and Chen 2011). Notably, the Shennongjia nature reserve contains 53 endemic genera and 1143 endemic species to China (Xie and Shen 2021; Xie et al. 2017). Endemic genera from Central‐East China mentioned in the results, mostly originating in this southeast region, are found to have dispersed to nature reserves both in the eastern and western parts of the QDM. Additionally, the Hengduan Mountains and the Zhongtiao‐Taihang Mountains are two additional centers for endemic genera distribution, with some species migrating into the QDM, leading to high biodiversity especially during the Quaternary glaciation and other ancient biological periods.

#### 4.2.2 Barrier Effect on Species Spread

In this study, we not only found that annual precipitation has the most significant impact on species distribution, but also revealed that absolute altitude does not solely determine whether a mountain acts as a corridor or a barrier for species dispersion. Within the QDM, the plant distribution pattern shows higher biodiversity at the easternmost and westernmost ends than in the central areas. We hypothesize that mountains act as corridors for species spreading in such areas as Baotianman in the east and Tangjiahe in the west, but as barriers in high‐altitude regions like Taibai Mountain and Gannan. This hypothesis is supported by other studies. For example, plants like *Actaea vaginata* (Wang 1979), *Kingdonia uniflora* and *Circaeaster agrestis* (Wu 1988), *Chrysosplenium uniflorum* and *C*. *griffithii* (Wang 1992) spread eastward from the Hengduan Mountains but stop at Taibai Mountain. Similarly, *Microula trichocarpa* and *Microula turbinata* spread eastward from western Sichuan but also stop in Mt. Taibai (Wang and Zhang 1994b). Chloroplast phylogeography studies have also identified the Wumengshan Mountains (in Southwest China) as barriers to gene flow between Lower Jinshajiang and Eastern Honghe/Nanpanjiang areas (Zhang et al. 2011). In these processes of species dispersal, Mt. Taibai also acts like a “isolated sky island.” We agree that high elevations can hinder species dispersal, with further quantitative analysis warranted.

By classifying the nature reserves in the QDM into five regions based on species similarity, we found that the elevation range within each region is similar, with the elevation range of less than 1000 m in maximum, minimum, and average height between nature reserves. However, when the elevation difference exceeds this threshold, species dispersal among different nature reserves is limited, leading to differences in plant flora richness between regions. This threshold of 1000 m is only a preliminary synthesis waiting for further testimony and more validation.

### 4.3 A New Perspective on Protecting Mountain Biodiversity

Inappropriate establishment of ecological corridors can lead to problems such as increased predator presence, invasive species, disease transmission, and unnecessary construction costs (Hilty et al. 2019). We propose a new approach to balance ecological preservation with human needs by using species distribution patterns to determine the natural ecological corridors within nature reserves in each region. Similar landform and elevation ranges can provide continuous migration corridors and habitats for species distribution, thereby effectively maintaining biodiversity and reducing corridor construction costs. Our findings indicate that within the same region, the differences in maximum, minimum, and average elevation differences among reserves should remain less than 1000 m. In contrast, elevation differences between reserves in different regions typically exceed 1000 m. In other words, when mountain range fluctuations surpass a certain threshold, biological exchange or dispersal may be significantly hindered.

Understanding how mountain structures influence species distribution is crucial for the rational development of the QDM and other mountainous areas. The planning of ecological corridors should consider species' natural distribution routes and extents. Future research should focus on refining analyses at finer spatial scales to ensure the long‐term ecological security of these ecologically significant regions.

## 5 Conclusion

The Qinling‐Daba Mountains serve as a crucial ecological corridor for plant dispersal between east (north) China and southwest China. This corridor provides similar habitats and continuous routes for species dispersal and significantly enhances biodiversity compared with the adjacent regions. High mountains such as Mt. Taibai (the main peak of Qinling Mts.) could be a barrier to species dispersal, which explains the high biodiversity in the easternmost and westernmost parts of the Qinling‐Daba Mountains but relatively low biodiversity in the central region.

## Author Contributions

**Ya Jiang:** conceptualization (equal), data curation (equal), formal analysis (equal), methodology (equal), validation (equal), writing – original draft (equal). **Baiping Zhang:** conceptualization (lead), investigation (equal), project administration (equal), writing – original draft (equal), writing – review and editing (equal). **Yonghui Yao:** methodology (equal), validation (equal), writing – review and editing (equal). **Jiayu Li:** data curation (equal), formal analysis (equal), methodology (supporting). **Junjie Liu:** investigation (equal), resources (equal).

## Conflicts of Interest

The authors declare no conflicts of interest.

## Supporting information


Data S1.

Table S1.

## Data Availability

The dataset of nature reserves used in this study, titled “A database of seed plants on taxonomy, geography, and ecology in the Qinling‐Daba Mountains and adjacent areas,” is publicly available on Figshare at https://doi.org/10.6084/m9.figshare.17919548. Introduced cultivated species were excluded by consulting *the Flora of China* database (available at www.iplant.cn/foc). Climate data from 89 climate stations for the period 2001–2017 was provided by http://www.resdc.cn. Additionally, Last Glacial Maximum climate data simulated by CCSM4 model (https://www.worldclim.org), terrain data were derived from the SRTM 90‐m resolution digital elevation model (available at https://lpdaac.usgs.gov). Code Availability: All analyses were done in R (v.4.4.0). The code used for the analyses is available upon request. Interested researchers can contact the corresponding author via email for access to the scripts.
